# Transcriptomic Analysis Reveals the Time-Dependent Mechanism of Antifungal Activity in *Bacillus velezensis* GHZJ-1

**DOI:** 10.3390/microorganisms14081809

**Published:** 2026-08-17

**Authors:** Wenji Chen, Yu Ni, Yuanyuan Bai, Mao Liu, Mingyao Xia, Bingyu Li

**Affiliations:** 1 Dalian Key Laboratory of Breeding, Reproduction and Aquaculture of Crustaceans, Dalian Ocean University, Dalian 116023, China; moxiaoshao@outlook.com (W.C.); niyu25@mails.ucas.ac.cn (Y.N.); newjourneyy@foxmail.com (Y.B.); liuliumaoa@outlook.com (M.L.); xiamingyao1@outlook.com (M.X.); 2 College of Aquaculture and Life Sciences, Dalian Ocean University, Dalian 116023, China

**Keywords:** *Bacillus velezensis*, antifungal activity, comparative transcriptomics, temporal regulation, biocontrol

## Abstract

Frequent outbreaks of bacterial and fungal diseases in aquaculture cause severe economic losses, making biological control using probiotics a crucial strategy. *Bacillus velezensis* synthesizes diverse antimicrobial secondary metabolites; however, its biocontrol potential is tightly regulated by environmental signals such as cultivation time. In this study, we investigated the effect of growing time on the antifungal activity of *B. velezensis* GHZJ-1, isolated from an aquatic environment. It was found that GHZJ-1 shows obvious antifungal activity against the aquatic pathogen *Metschnikowia bicuspidata* upon 48 h growth but no such activity for 24 h via the agar-diffusion method. We further compared the transcriptomes of GHZJ-1 cells collected at 24 h and 48 h through RNA-Seq. Our results revealed that compared with 24 h, 1821 genes were differentially expressed at 48 h, with 903 upregulated and 918 downregulated. Downregulated genes were enriched in primary metabolic pathways (e.g., ribosome assembly and carbon metabolism), whereas upregulated genes were enriched in secondary metabolite biosynthesis and transmembrane transport. Importantly, 35 upregulated genes directly associated with antimicrobial activity were identified, notably including the ones encoding core elements of a large polyketide synthase (e.g., *pksN*, log_2_FC = 2.24), the petrobactin siderophore system (highest log_2_FC = 3.39), and various antimicrobial peptide export systems. Furthermore, the *degU* gene was activated at 48 h. These data suggest that facing continuously increased environmental stress over time, GHZJ-1 undergoes global transcriptional reprogramming and resource reallocation, downregulating basal metabolism to construct a synergistic antagonistic system coupling chemical defense with nutritional competition. This study elucidates the time-dependent mechanism for antifungal activity in GHZJ-1, providing a molecular theoretical basis for its green biocontrol application in aquaculture diseases caused by fungi.

## 1. Introduction

With the continuous expansion of intensive agricultural production worldwide, epidemic diseases caused by pathogenic bacteria and fungi have become increasingly frequent, resulting in massive economic losses [1]. Traditional chemical drugs and antibiotics once provided efficient solutions for modern agricultural disease management; however, in fields such as livestock, aquaculture, and crop protection, their prolonged and excessive application has led to the accumulation of pathogen resistance, drug residues in the food chain, and ecological disruption, posing global public health and environmental security challenges [2,3]. Within this context, biological control strategies centered on probiotics have emerged as a vital approach to replacing antibiotics and achieving sustainable agricultural production owing to their high safety, low risk of inducing resistance, and environmental friendliness [4].

Among various candidate probiotics, *Bacillus* spp. have become a research hotspot in the biocontrol of agricultural and aquaculture diseases due to their high stress resistance, ability to form highly resilient endospores, outstanding colonization capacity, and prolific production of metabolites [5]. Specifically, *Bacillus velezensis* has garnered widespread attention for its broad-spectrum antagonistic activity, as it can synthesize dozens of antimicrobial secondary metabolites, including lipopeptides, polyketides, siderophores, and bacteriocins [6,7]. This metabolic diversity confers vast biocontrol application potential across multiple sectors, including livestock farming, aquaculture, and crop disease management [8].

The biocontrol potential of *B. velezensis* fundamentally relies on its rich repertoire of secondary metabolites [9]. Upon reaching a specific cell density threshold, quorum-sensing systems (e.g., ComPA) and two-component regulatory systems are activated. Acting as crucial signaling nodes, these systems coordinate with the Spo0A cascade to globally regulate the biosynthesis of broad-spectrum antimicrobial compounds [10,11]. By synthesizing high-affinity siderophores and massive polyketide synthase (PKS) complexes, *Bacillus* effectively inhibits pathogen colonization and proliferation, thereby acquiring a superior competitive advantage [12]. Currently, research elucidating these antimicrobial mechanisms predominantly focuses on the functional identification of individual antimicrobial peptide genes or the fermentation optimization of specific metabolites in model strains such as FZB42 [13].

However, the biosynthesis of the aforementioned antimicrobial substances is not constitutive; rather, it is subjected to strict temporal regulation governed by cultivation time, nutrient conditions, and environmental signals [14]. Confronted with progressive nutrient depletion as cultivation extends, *Bacillus* must execute precise resource reallocation and metabolic trade-offs between basal cell proliferation and the synthesis of secondary defense compounds [15]. Previous studies indicate that the substantial accumulation of secondary metabolites in *Bacillus* typically occurs within a specific physiological window, spanning from the late logarithmic phase to the stationary phase [16]. Given the close phylogenetic relationship between *B. velezensis* and *Bacillus subtilis*, these temporal regulatory networks are highly likely to be conserved. Nevertheless, how “cultivation time” acts as a key parameter to globally orchestrate this process lacks systematic transcriptomic evidence, making transcriptomic analysis an essential tool with which to decipher this resource allocation and defense activation network [17].

In the previous research, we isolated *B. velezensis* GHZJ-1 and characterized its antimicrobial activity against *Enterobacter bugandensis*, a Gram-negative bacterium [18]. This study investigates the impact of cultivation time on antifungal activity against *Metschnikowia bicuspidata*, an emerging yeast pathogen causing the “milky disease” in Chinese mitten crabs [19], and the transcriptome of GHZJ-1 to provide molecular evidence for its mechanisms for antifungal substance synthesis. By identifying differentially expressed genes (DEGs) and performing GO/KEGG functional enrichment, we systematically mapped the dynamic gene expression shifts over the growth period—from 24 h to 48 h—identifying key metabolic pathways and genes contributing to the antifungal activity of GHZJ-1. This research not only offers novel insights into the environmental adaptation strategies (such as nutritional competition and stress responses) of *Bacillus* but also provides theoretical guidance for optimizing industrial fermentation and facilitating the genetic modification of *Bacillus* strains for high-yield bioactive substance production.

## 2. Materials and Methods

### 2.1. Strains and Culture Conditions

The *B. velezensis* GHZJ-1 strain used in this study was collected from our previous research. Routine cultivation of the strain was performed in YT broth medium [20 g/L tryptone (Guangdong Huankai Microbial Sci. & Tech. Co., Ltd., Guangzhou, China), 10 g/L yeast extract (Guangdong Huankai Microbial Sci. & Tech. Co., Ltd.)], which is salt-free, at 30 °C with shaking at 220 rpm.

The *M. bicuspidata* GHI202204 strain was a lab collection and routinely grown in YPD agar medium (Macklin Biochemical Co., Ltd., Shanghai, China) at 30 °C.

### 2.2. Preparation of GHZJ-1 Supernatant Samples at Different Cultivation Times

The GHZJ-1 strain, preserved at −80 °C, was streaked into nutrient agar (Guangdong Huankai Microbial Sci. & Tech. Co., Ltd.) and grown overnight at 30 °C. A single colony was then inoculated into 2 mL of YT broth medium and incubated overnight at 30 °C with shaking at 220 rpm. The overnight culture was transferred at a 1:100 ratio into 20 mL of fresh YT broth medium and cultured under the same conditions for 24 h and 48 h, respectively. Centrifugation at 12,000 rpm for 3 min was used to harvest bacterial cells for total RNA extraction, and to obtain culture supernatant for agar well diffusion assays. The supernatant was filter-sterilized using 0.22 μm filters [Sangon Biotech (Shanghai) Co., Ltd., Shanghai, China]. Three independent experiments were set up for each cultivation time.

### 2.3. Agar Well Diffusion Assay for Antifungal Activity

The agar well diffusion method was employed to determine the antifungal activity of the GHZJ-1 culture supernatant against *M. bicuspidata*. Briefly, colonies of *M. bicuspidata* were suspended in sterile saline to prepare a cell suspension with a density of approximately 1 × 10^8^ cells/mL. A sterile cotton swab was used to uniformly inoculate the suspension onto the surface of YPD agar plates (Macklin Biochemical Co., Ltd.). After the plate surfaces dried, wells were sterilely punched into the yeast-inoculated agar. Subsequently, culture supernatants from different cultivation times (24 h and 48 h) of GHZJ-1 were added to the respective wells. Finally, the plates were incubated statically at 30 °C, and the formation of inhibition zones was observed and recorded at distinct time points.

### 2.4. Total RNA Extraction

Cell pellets of GHZJ-1 (see Section 2.2) were subjected to RNA extraction. Total bacterial RNA was extracted using the Bacterial RNA Kit R6950 (Omega Bio-tek, Inc., Norcross, GA, USA) following the manufacturer’s instructions. The main procedures included lysozyme treatment for cell wall disruption, mechanical disruption using micro glass beads, purification via an RNA spin column, and final elution to obtain the total RNA solution. All RNA samples (6 in total) were quality-checked prior to subsequent library construction and sequencing.

### 2.5. RNA-Seq

The concentration, total amount, and integrity of the RNA samples were precisely assessed using an Agilent 2100 Bioanalyzer (Agilent Technologies, Santa Clara, CA, USA). Upon confirming their qualification, the strand-specific library was constructed. The main workflow included: enriching mRNA by depleting ribosomal RNA; randomly fragmenting the mRNA into short sequences using Fragmentation Buffer; synthesizing first-strand cDNA using random hexamer primers; synthesizing second-strand cDNA using dUTP instead of dTTP; end repair, A-tailing, adapter ligation, size selection, and PCR amplification. The libraries passed quality control using a Qubit 2.0 Fluorometer (Thermo Fisher Scientific, Waltham, MA, USA) and were subsequently subjected to high-throughput paired-end sequencing on an Illumina platform (Illumina, San Diego, CA, USA) by Novogene Co., Ltd. (Beijing, China).

### 2.6. Transcriptome Data Analysis

To ensure the accuracy of downstream analyses, the raw sequencing data were first strictly filtered to obtain clean data by removing adapter-containing reads, poly-N reads, and low-quality reads (where bases with Qphred ≤ 5 constitute more than 50% of the total read length). Subsequently, Bowtie2 v2.5.4 (Johns Hopkins Bloomberg School of Public Health, Baltimore, MD, USA) [20] was used to align the clean data to the reference genome of *B. velezensis* GHZJ-1 (NCBI Accession No.: CP184280.1). Following alignment, featureCounts v2.0.6 (Walter and Eliza Hall Institute of Medical Research, Parkville, Australia) [21] was utilized to calculate the read counts mapped to each gene, which were then converted into FPKM values to eliminate the effects of gene length and sequencing depth. The biological replicate correlations among samples were evaluated by calculating the Pearson correlation coefficient based on genome-wide log_2_(FPKM + 1) transformed values. Differential expression analysis was performed using the DESeq2 R package v1.42.0 (R Foundation for Statistical Computing, Vienna, Austria) [22] based on the negative binomial distribution model. To control the false discovery rate, *p*-values were adjusted using the Benjamini-Hochberg approach (*p*). In this study, |log_2_(FoldChange)| ≥ 1 and *p* ≤ 0.05 were utilized as the thresholds for identifying significantly differentially expressed genes (DEGs). Finally, the clusterProfiler R package v4.8.1 (R Foundation for Statistical Computing, Vienna, Austria) [23] was used to perform Gene Ontology (GO) [24] and Kyoto Encyclopedia of Genes and Genomes (KEGG) [25] pathway enrichment analyses on the screened DEGs. The functional annotations of the differentially expressed genes were performed based on the Swiss-Prot database.

## 3. Results

### 3.1. Effect of Cultivation Time on the Antifungal Activity of GHZJ-1

**Figure 1:**
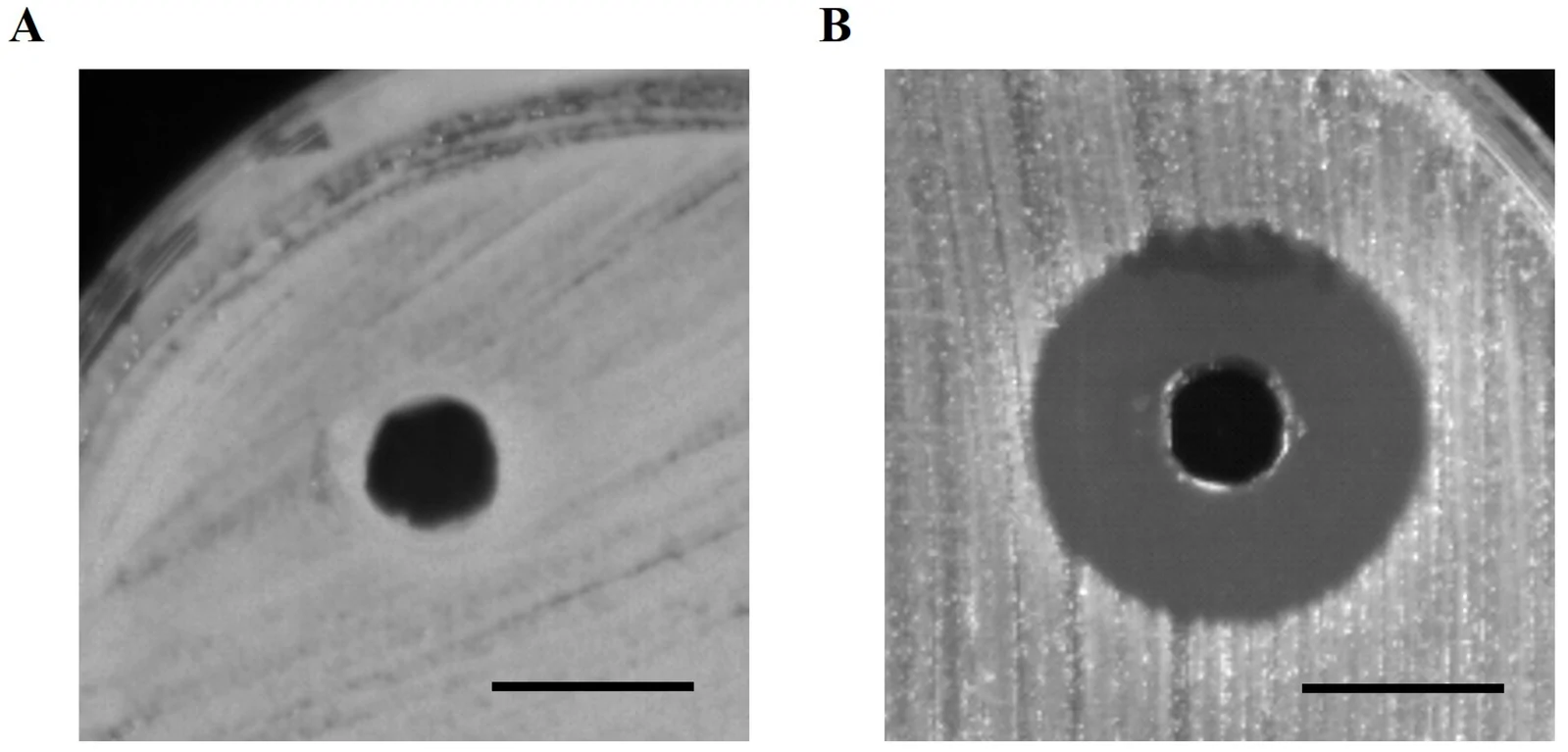
Inhibitory effects of GHZJ-1 culture supernatants from different cultivation times against *M. bicuspidata* (agar well diffusion assay). (**A**) YT broth culture supernatant (24 h); (**B**) YT broth culture supernatant (48 h). The black scale bars represent 10 mm; the results shown are representative of 3 independent experiments.

In our previous study, GHZJ-1 was isolated from the tailwater recycling pond of a crab and shrimp aquaculture base. This strain showed antifungal activity during our initial screening test, in which we grew it and the pathogen yeast *M. bicuspidata* together in YPD agar, a classic medium for the cultivation of yeasts. Given that YPD agar is salt-free, and that biomass accumulation under salt-free conditions is significantly higher than that under high-salt conditions in certain *Bacillus* strains [26], we used a salt-free medium, YT broth (see Section 2), to grow GHZJ-1 properly without impairing its production of antifungal metabolites in this study. To evaluate the effect of cultivation time on the antifungal activity of GHZJ-1, the agar well diffusion method was employed to test the culture supernatants, obtained at different cultivation times, against *M. bicuspidata* (see Section 2). The results demonstrated that the supernatant from the 24-h culture failed to produce a distinct inhibition zone (Figure 1A), indicating that the strain had not yet accumulated sufficient active substances to inhibit the yeast at this stage. However, when the cultivation time was extended to 48 h, a clear yeast-inhibition zone (with a diameter of ~20 mm) formed around the fermentation supernatant (Figure 1B), demonstrating a significant enhancement in its antifungal activity.

### 3.2. Quality Assessment of RNA-Seq and Overall Transcriptomic Differences

To ensure the reliability of the transcriptome data, the sequencing data quality of the 6 samples was first assessed. The number of effective reads for each sample ranged from 14.44 million to 16.43 million, the Q30 base percentage was consistently above 97%, and the GC content ranged from 46.8% to 47.9% (Table 1). Combined with the RNA integrity assessment results (all RIN values ≥ 7.6), the sequencing data quality of all samples met the requirements for subsequent downstream analyses.

**Figure 2:**
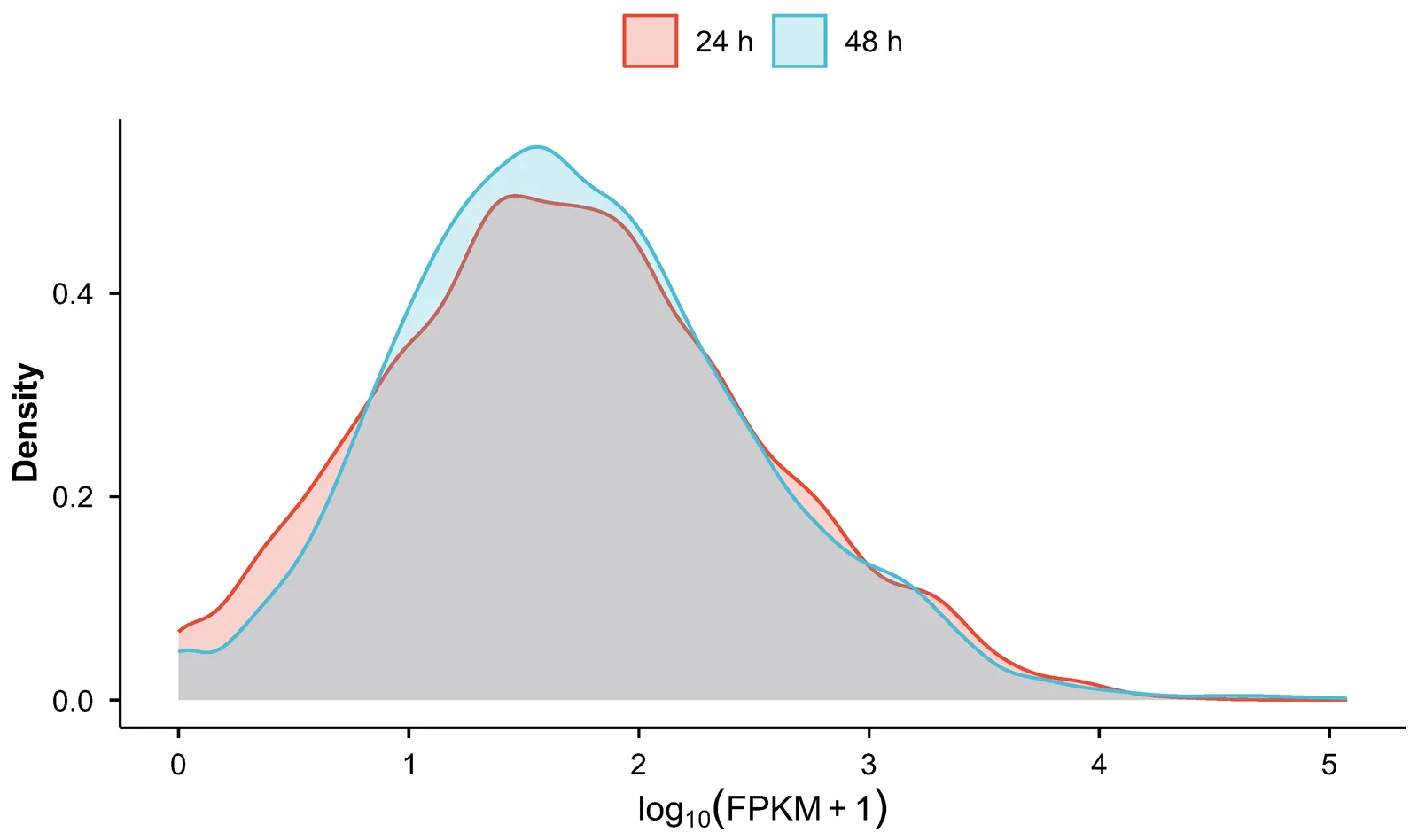
FPKM density distribution of gene expression levels in 24-h and 48-h cultures. Based on the FPKM values of all 4222 expressed genes, the x-axis represents the log_10_(FPKM + 1) transformed expression levels, and the y-axis represents the gene density. The red curve represents the 24-h group (*n* = 3), and the blue curve represents the 48-h group (*n* = 3).

To evaluate the global transcriptional state of the cells at different cultivation times, a gene expression density distribution map was generated based on the FPKM values of all 4222 expressed genes (Figure 2). On a macroscopic scale, the overall overlap of the expression abundance curves between the 24-h and 48-h samples was high, presenting a typical skewed unimodal distribution, which indicates reliable sequencing data quality and good global comparability between the two groups. However, the detailed morphology of the density curves revealed dynamic differences at the transcriptional level between the two cultivation stages. Specifically, the 24-h group exhibited a relatively higher gene density in the low-expression range (x-axis 0–1.0). This may be associated with the more active growth state of the cells during the early cultivation phase, during which a broader range of background gene transcription was maintained. In contrast, when the cultivation time was extended to 48 h, the peak value of its main expression peak (x-axis 1.5–2.0) significantly increased and became more concentrated. This rightward shift and convergence of the peak indicate that, as cultivation progressed, the cells underwent significant transcriptional resource reallocation: the bacteria likely downregulated the widespread expression of certain non-essential genes, redirecting the transcriptional machinery and energy toward the high-abundance expression of specific functional gene clusters (e.g., secondary metabolic pathways). This convergence and shift in the global expression pattern provide preliminary transcriptomic evidence of the differences in antagonistic activity observed at 48 h in this study.

**Figure 3:**
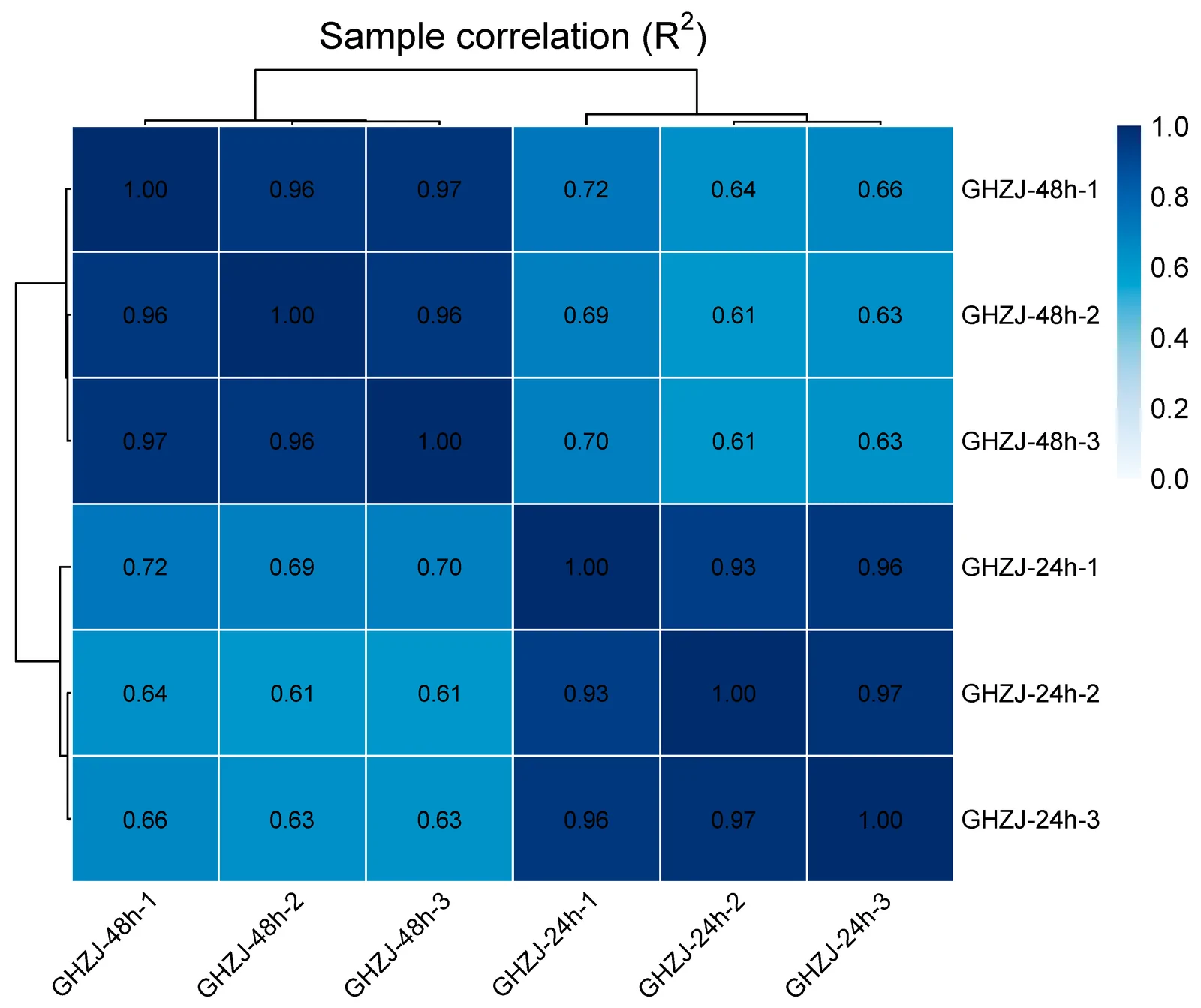
Pearson correlation heatmap of gene expression levels among samples. Based on the log_2_(FPKM + 1) values of all 4222 expressed genes, the square of the Pearson correlation coefficient (R^2^) between pairwise samples was calculated. The color intensity represents the correlation strength, with darker blue indicating higher R^2^ values. The numbers within the grid represent the exact R^2^ values (rounded to two decimal places). Hierarchical clustering was performed using the complete linkage method.

To assess the reliability of independent experiments and quantify transcriptomic differences across different cultivation stages, the square of the Pearson correlation coefficient (R^2^) among all pairwise samples was calculated based on the log_2_(FPKM + 1) transformed values of all 4222 expressed genes, followed by hierarchical clustering using the complete linkage method (Figure 3). The results showed that the R^2^ values among the 3 independent experiments within the 24-h group ranged from 0.93 to 0.97, and within the 48-h group from 0.96 to 0.97, indicating excellent intra-group reproducibility and highly reliable experimental operations for both groups. In contrast, the R^2^ values between samples from the 24-h and 48-h groups decreased to 0.61–0.72, and the hierarchical clustering tree distinctly separated the 6 samples into two independent branches corresponding to 24 h and 48 h. These results indicate that during the cultivation process from 24 h to 48 h, the overall transcriptome of GHZJ-1 underwent significant reprogramming, with inter-group differences vastly exceeding intra-group differences, thereby providing a robust data foundation for the subsequent screening of differentially expressed genes.

### 3.3. Screening and Functional Enrichment Analysis of DEGs

**Figure 4:**
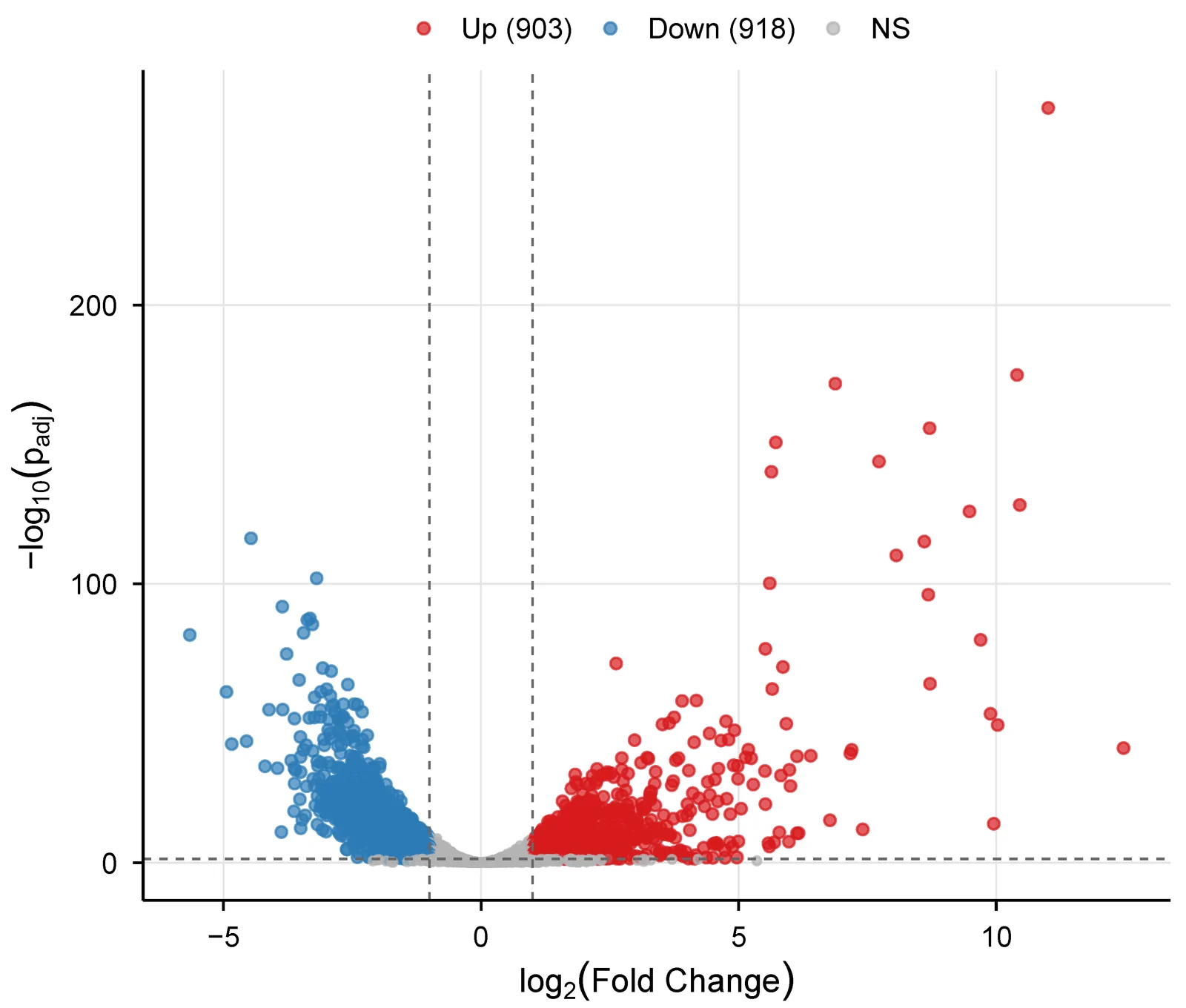
Volcano plot of DEGs between 24-h and 48-h cultivation conditions. The x-axis represents the log_2_(Fold Change), and the y-axis represents the −log_10_(*p*). Red dots represent significantly upregulated genes (*p* ≤ 0.05, log_2_FC ≥ 1), blue dots represent significantly downregulated genes (*p* ≤ 0.05, log_2_FC ≤ −1), and gray dots represent genes with no significant difference. A total of 1821 DEGs were identified, including 903 upregulated and 918 downregulated genes. The dashed lines indicate the significance thresholds (*p* ≤ 0.05, |log_2_FC| ≥ 1).

To clarify the specific effects of cultivation time on gene expression in GHZJ-1, DEGs were screened using thresholds of |log_2_(FoldChange)| ≥ 1 and *p* ≤ 0.05. As shown in Figure 4, compared to the 24-h group, a total of 1821 DEGs were identified in the 48-h group, comprising 903 upregulated and 918 downregulated genes. The volcano plot exhibited a distinctly symmetrical distribution of DEGs, indicating that the cells underwent drastic transcriptional regulatory fluctuations during the cultivation period from 24 h to 48 h, with the numbers of up- and downregulated genes being approximately equal.

Furthermore, the gene (ACNBEW_16770) encoding the global regulator DegU was significantly upregulated at 48 h (log_2_FC = 1.52946036, *p* = 1.0 × 1.03^−13^). In contrast, the 2 genes (ACNBEW_06215, ACNBEW_14735) encoding the receptor kinase ComP of the ComPA quorum-sensing system exhibited differential regulation: one was significantly downregulated (log_2_FC = −1.13, *p* = 5.89 × 10^−6^), while the other showed a downregulation trend but did not reach the significance threshold (log_2_FC = −0.41, *p* = 0.226).

**Figure 5:**
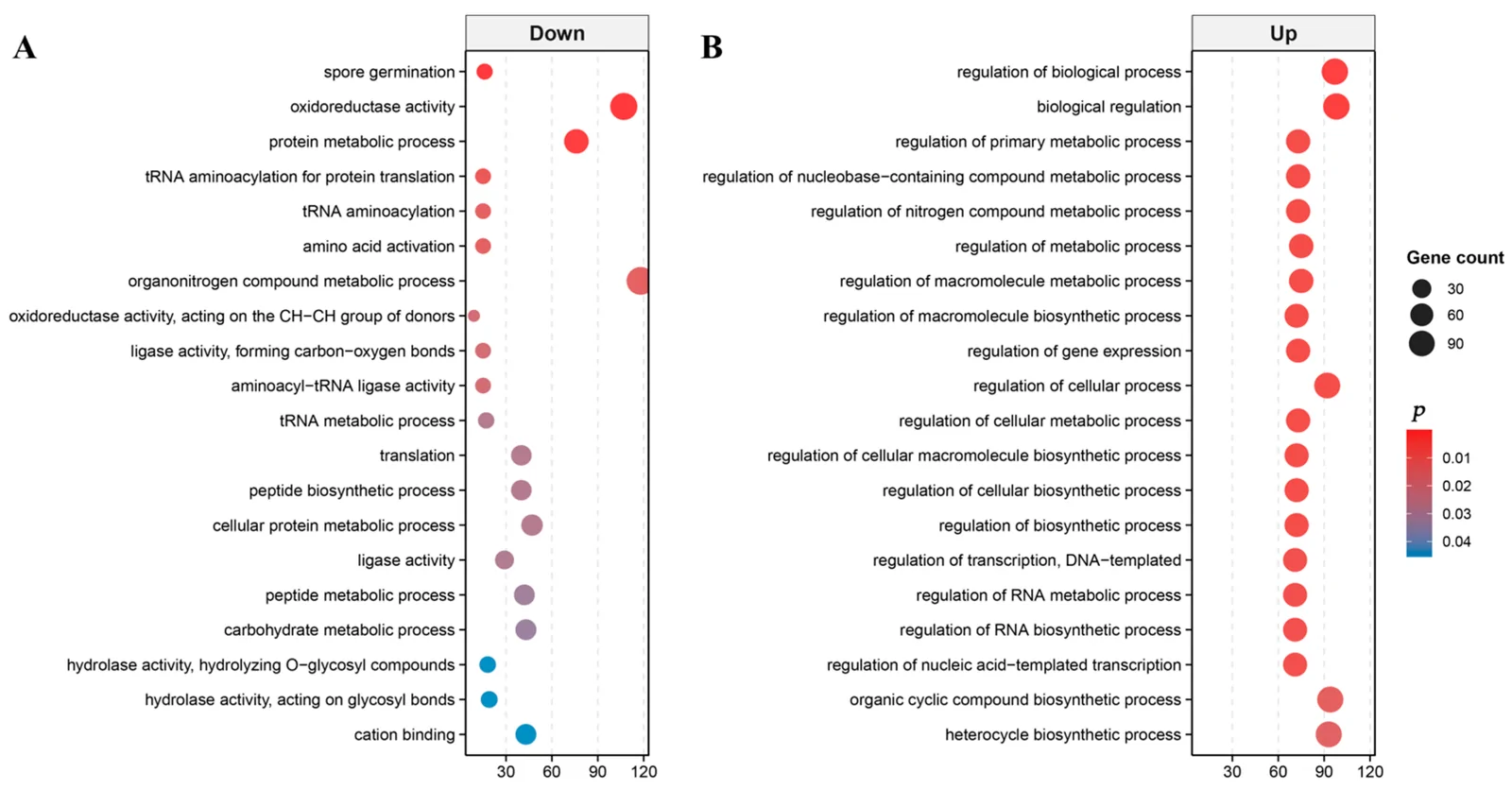
GO functional enrichment analysis of differentially expressed genes. (**A**) Enriched GO terms of the downregulated DEGs. (**B**) Enriched GO terms of the upregulated DEGs. The top 20 most significant terms with *p* < 0.05 are displayed for each. The x-axis represents the number of DEGs enriched in each GO term, bubble size represents the number of genes, and color represents the significance level (*p*), with darker red indicating higher significance.

GO functional enrichment analysis was performed for the upregulated and downregulated DEGs, respectively (Figure 5). The results indicated that the downregulated genes were highly enriched in pathways related to foundational life activities, including ribosome assembly, amino acid metabolism, and primary energy conversion (Figure 5A). In contrast, the genes significantly upregulated at 48 h were mainly enriched in biological processes such as secondary metabolite biosynthesis, regulation of biosynthetic processes, and cellular response to stress (Figure 5B).

**Figure 6:**
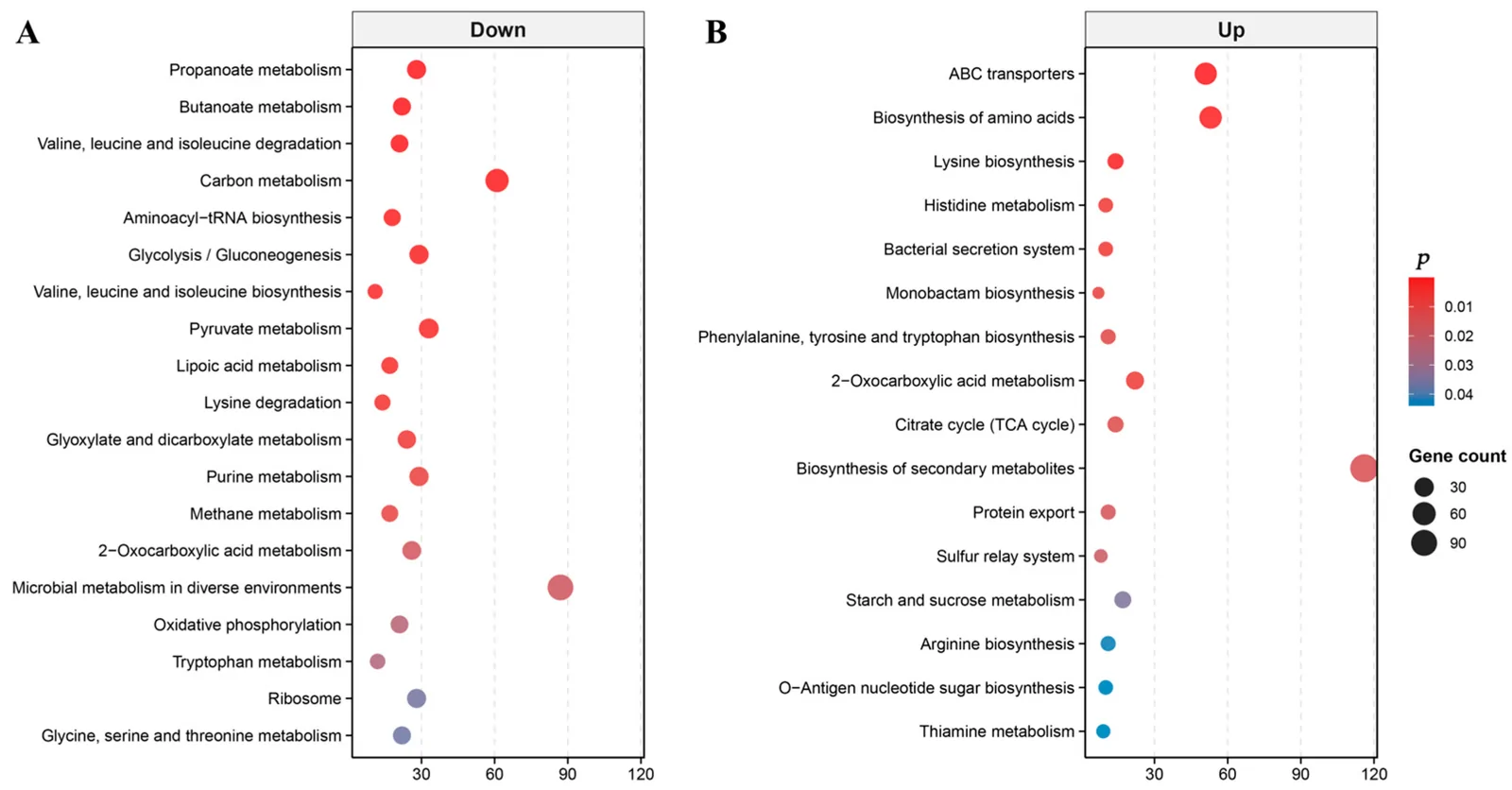
KEGG pathway enrichment analysis of differentially expressed genes. (**A**) Enriched KEGG pathways of the downregulated DEGs. (**B**) Enriched KEGG pathways of the upregulated DEGs. The top 20 pathways with *p* < 0.05 are displayed for each. The x-axis represents the number of DEGs enriched in each pathway, bubble size represents the number of genes, and color represents the significance level (*p*), with darker red indicating higher significance.

KEGG pathway enrichment analysis further detailed the metabolic networks involving the DEGs (Figure 6). The downregulated genes were significantly enriched in primary energy metabolic networks such as carbon metabolism and Glycolysis/Gluconeogenesis, as well as foundational translation pathways including Aminoacyl-tRNA biosynthesis and Ribosome (Figure 6A). Concurrently, among the upregulated genes, the core pathway exhibiting the most significant enrichment and containing the largest number of genes was Biosynthesis of secondary metabolites (Figure 6B). Additionally, pathways such as ABC transporters and Bacterial secretion system were also significantly enriched (Figure 6B).

### 3.4. Transcriptional Activation of Genes Related to Secondary Metabolites

Based on the DEG screening results, this study focused on analyzing the secondary metabolite biosynthesis gene clusters directly associated with antimicrobial activity. As shown in Table 2, a total of 19 polyketide synthesis-related genes were significantly upregulated at 48 h. Notably, the core members of the PKS gene cluster, *pksN*, *pksL*, and *pksJ*, exhibited particularly significant upregulation, with the maximum log_2_FC value reaching 2.24. Concurrently, genes involved in the regulation of polyketide biosynthesis, such as *baeC*, *baeD*, and *baeE*, were also upregulated synchronously.

Furthermore, genes associated with siderophore biosynthesis and transport underwent intense transcriptional activation. The *yclN*, *yclO*, and *yclP* genes responsible for petrobactin transport were significantly upregulated at 48 h, with the log_2_FC of *yclN* reaching 3.39. Concurrently, genes related to catecholate siderophore biosynthesis (*dhbA/B/C/E*) also exhibited an upregulation trend, suggesting that iron competition mechanisms may be involved in the antagonistic process. Regarding antimicrobial peptides, genes associated with microcin secretion (*mchF*), bacitracin transport (*bcrA*), and subtilosin biosynthesis (*albG*) were all significantly upregulated.

## 4. Discussion

This study demonstrated that the 48-h culture supernatant of *B. velezensis* GHZJ-1 exhibited significant antagonistic activity against the pathogenic yeast *M*. *bicuspidata*, whereas the 24-h supernatant showed no such effect (Figure 1). Furthermore, the supernatant of this strain also possesses antagonistic activity against Gram-negative bacteria, and this activity exhibits the same time-dependent pattern as observed with *M. bicuspidata* [18]. Taken together, GHZJ-1 is likely to be capable of synthesizing metabolites with broad-spectrum antimicrobial potential, and their synthesis requires activation by specific cultivation durations. Broad-spectrum antimicrobial activity typically involves multidimensional chemical defense mechanisms and complex secondary metabolites, making it difficult for traditional phenotypic observations or single-target gene/metabolite analyses to comprehensively reveal their underlying regulatory logic [27,28]. The core advantage of comparative transcriptomics lies in its ability to systematically capture the global expression profile of bacteria at a specific physiological stage [29]. Therefore, utilizing transcriptomic approaches, this study aimed to overcome the limitations of single-mechanism analyses and systematically investigate the global transcriptional reprogramming characteristics of GHZJ-1 driven by the growth condition of “cultivation time”, thereby providing a molecular explanation for its broad-spectrum antagonistic effects, including against notorious aquatic fungi like *M*. *bicuspidata* [30].

From 24 h to 48 h, the strain underwent global transcriptional reprogramming, visually reflected in the rightward shift of the main gene expression density peak (Figure 2), the decrease in the inter-group Pearson correlation coefficient (Figure 3), and the identification of 1821 DEGs (Figure 4). These transcriptional fluctuations mark a shift in the physiological focus of the bacteria: transitioning from the comparatively early growth phase at 24 h, which might not largely activate secondary metabolite synthesis, to the late growth phase at 48 h, which focuses on the accumulation of secondary metabolites. This temporal reconstruction of the transcriptional profile provides transcriptomic evidence for the enhanced biocontrol antifungal potential of GHZJ-1 during the late phase, at least later than 24 h post inoculation.

The transition from primary to secondary metabolism is governed by complex gene cascade regulatory networks, and environmental factors such as nutrient depletion and the concentration thresholds of quorum-sensing signaling molecules are typically the key triggers for this transcriptional program [31]. In *Bacillus*, the Spo0A phosphorylation cascade serves as a central hub connecting environmental nutritional status with genomic expression reprogramming, and its activation can relieve transcriptional repression on numerous secondary metabolite clusters [32]. And yet, expression of *spo0A* remained unchanged (log_2_FC = −0.004317248, *p* = 0.986436368) in this study. Intriguingly, the transcriptional regulatory factor gene *degU* exhibited highly significant upregulation at 48 h, and recent research has confirmed the core role of the DegSU system in regulating extracellular proteases and secondary metabolism [33]. In contrast, one gene encoding the quorum-sensing receptor protein ComP (*comP*) was significantly downregulated at this stage. ComPA is a well-known quorum-sensing system in *Bacillus* [34]. As cell density increases, extracellular signaling molecules accumulate and activate the membrane receptor ComP, which subsequently phosphorylates the response regulator ComA to trigger downstream secondary metabolism [35]. Therefore, our data regarding *comP* seem to be not in accordance with the antifungal activity at 48 h. However, secondary metabolism regulation in *Bacillus* is a complex network involving Spo0A, DegSU and ComPA [36]. In addition, RNA-Seq results only reveal the expression of genes at the level of mRNA; they cannot fully represent the cellular level and actual activity of their respective products. Taken together, more genetic studies are required to elucidate the effects of these global regulatory systems on secondary metabolite synthesis in GHZJ-1; we will address this in our future research.

Transcriptomic data suggested that after 48 h of cultivation, GHZJ-1 constructed a multidimensional antagonistic mechanism synergizing direct chemical inhibition and indirect nutritional competition at the gene expression level. Regarding direct chemical inhibition, as shown in Table 2, the strain coordinately upregulated a large polyketide synthase (PKS) gene cluster comprising 19 genes, covering the core synthases required for carbon backbone synthesis (*pksN*, *pksL*, *pksJ*, *pksM*), acyl carrier proteins (*acpK*), and a series of structural modification enzymes (*pksH*, *pksI*, *baeC*, *baeD*, *baeE*, *baeG*). Among these, the expression levels of the core elements (e.g., *pksN* and *pksL*) were extremely significantly elevated. In *B. velezensis*, large PKS gene clusters are responsible for synthesizing broad-spectrum antimicrobial polyketides such as bacillaene and difficidin, which exert inhibitory activities by blocking bacterial protein synthesis [37,38]. Meanwhile, genes encoding multiple antimicrobial peptide transmembrane transport and synthesis systems were also significantly upregulated, including the bacitracin transport ATP-binding protein (*bcrA*), export permease (*bceB*), microcin secretion protein (*mchF*), and subtilosin biosynthesis protein (*albG*). Particularly noteworthy is the significant upregulation of *mchF*, a gene classically linked to microcin secretion in *Escherichia coli*. Although microcin biosynthesis was traditionally considered restricted to Gram-negative bacteria, recent studies have characterized microcin-like antibiotics and their corresponding export systems in *Bacillus* species, such as Bsu-McC in *B. subtilis* [39]. Therefore, the annotation of *mchF* in GHZJ-1 likely arises from substantial structural and functional conservation within the antimicrobial peptide-associated ABC transporter superfamily. The marked transcriptional activation of this gene suggests that GHZJ-1 may harness a homologous secretion system, potentially acquired through horizontal gene transfer, to mediate the efficient efflux of microcin-like peptides or other bacteriocins, thereby antagonizing *M. bicuspidata*. Notably, unlike the typical biocontrol strain *B. velezensis* FZB42, which primarily relies on an early defense strategy involving lipopeptide antibiotics, GHZJ-1 exhibited a late-stage antagonistic characteristic centered on polyketide synthesis and siderophore transport at 48 h [40]. Furthermore, a comparative transcriptomic study discovered that the PKS gene cluster in *B. velezensis* LZN01 was similarly significantly upregulated under optimized fermentation conditions, indicating that the temporal activation of the polyketide synthase gene cluster might be a conserved regulatory feature in *B. velezensis*. However, it is worth noting that the Non-Ribosomal Peptide Synthetase (NRPS) gene cluster was also significantly activated in LZN01, whereas the response of the NRPS system in GHZJ-1 at 48 h was relatively limited, and the upregulation of its antimicrobial peptide-related genes was mainly concentrated in specific transport proteins and secretion systems (Table 2). This implies that different strains may exhibit strain-specific regulatory strategies for secondary metabolites [41]. As correctly suggested by comparative genomics, this distinctive responsiveness is fundamentally driven by the strain-specific genomic information. Through evolutionary adaptation and horizontal gene transfer, different *B. velezensis* strains harbor highly customized repertoires of biosynthetic gene clusters and distinct regulatory promoter architectures [42]. Consequently, identical environmental signals can trigger divergent transcriptomic cascades and resource allocations across different strains.

In terms of competitive nutritional acquisition, GHZJ-1 demonstrated high-intensity environmental iron-affinity uptake characteristics. Its siderophore system underwent drastic transcriptional activation at 48 h, among which the permease gene *yclN*, responsible for petrobactin transport, showed the most significant upregulation, while its complementary ATP-binding protein (*yclP*) and substrate-binding protein (*yclQ*) were also synergistically activated. The DHB gene cluster (*dhbA/B/C/E*), responsible for synthesizing catecholate siderophores, exhibited a consistent upregulation trend. As a high-affinity catecholate siderophore, the synthesis and secretion of petrobactin can effectively chelate trace amounts of free Fe^3+^ in the environment [43]. In oxygen-rich, slightly alkaline aquatic environments, free ferric iron (Fe^3+^) readily forms insoluble precipitates, resulting in extremely low bioavailability [44]. Siderophore-mediated nutritional immunity has been recognized as one of the important antagonistic strategies within microbial communities [45]. By highly expressing the petrobactin siderophore system, GHZJ-1 can effectively deprive coexisting pathogenic microorganisms of available iron sources, thereby constructing a competitive defense barrier based on nutritional exclusion. It has been described that efficient iron uptake is crucial for the stress response and virulence of pathogenic yeasts including *M*. *bicuspidata* and *Candida* spp. [46,47]. The robust siderophore production by GHZJ-1 can create a severely iron-depleted microenvironment. This intense nutritional competition can deprive *M. bicuspidata* of essential iron resources, thereby impairing its stress resistance, attenuating its virulence, and ultimately suppressing its proliferation within the hosts.

The biosynthesis of macromolecular PKS enzyme complexes and high-affinity siderophores heavily relies on massive inputs of intracellular free ATP and carbon-nitrogen backbone substrates. In resource-limited environments, when microorganisms allocate limited carbon sources and energy to the synthesis of secondary metabolites, it inevitably comes at the expense of reducing basal metabolic investments related to proliferation [48]. As shown in Figure 5 and Figure 6, GO and KEGG enrichment analyses in this study indicated that the networks for ribosome assembly, amino acid biosynthesis, and basal carbon metabolism were significantly downregulated overall at 48 h, reflecting a resource reallocation adaptive strategy in response to environmental stress. In *B. subtilis*, the metabolic characteristics of the stationary phase have been confirmed to include the significant downregulation of ribosomal protein expression and the overall suppression of carbon metabolic pathways, which highly aligns with the downregulation pattern observed in this study [49]. By curtailing material investments in the foundational transcription-translation machinery and primary energy metabolism, the bacteria can reallocate restricted endogenous carbon backbones and energy carriers to compensate for the energy deficit caused by the synthesis of macromolecular secondary metabolites. This regulation reveals the adaptation mechanism of GHZJ-1 in nutrient-limited habitats [50].

This study systematically evaluated the biocontrol potential of GHZJ-1 based on transcriptomic correlation analysis; however, the cascade amplification effect of transcript abundance means it is not completely equivalent to the absolute concentration of the final targeted metabolites. Future research plans to combine untargeted or targeted metabolomics technologies to identify the structural compositions of substances in the 48-h culture supernatant, which will further clarify the exact chemical nature of its antimicrobial activity.

Despite the insights gained from our transcriptomic analysis, several technical limitations regarding DEG screening and KEGG enrichment must be acknowledged. First, the analysis relies on bulk RNA-Seq at discrete time points (24 h and 48 h), which captures the average population-level expression but inherently masks the continuous temporal dynamics and single-cell phenotypic heterogeneity of the bacterial population. Second, DEG screening based on strict statistical thresholds may exclude genes with subtle but biologically crucial expression changes. Lastly, KEGG pathway enrichment is intrinsically constrained by the completeness of current database annotations. Since existing databases are often biased toward well-studied model organisms, novel or uncharacterized secondary metabolic pathways specific to *B. velezensis* GHZJ-1 might be overlooked or misclassified. Future studies incorporating time-series transcriptomics and single-cell RNA sequencing are needed to capture a more comprehensive and highly resolved regulatory landscape.

## 5. Conclusions

Through comparative transcriptomic analysis, this study systematically reveals that cultivation time is a key factor regulating the antifungal activity of *B*. *velezensis* GHZJ-1 against *M*. *bicuspidata*. From 24 h to 48 h, the strain underwent global transcriptional reprogramming, with over 40% of its genes exhibiting significant differential expression, shifting its metabolic focus from basal proliferation to secondary metabolite biosynthesis. At 48 h, a large polyketide synthase (PKS) gene cluster containing 19 members, the petrobactin catecholate siderophore biosynthesis and transport system, and various antimicrobial peptide export systems all exhibited significant transcriptional activation. Simultaneously, ribosome assembly and basal carbon metabolic pathways were globally downregulated, reflecting a resource reallocation strategy between “growth” and “defense.” These findings elucidate the molecular basis underlying the enhancement of GHZJ-1’s antagonistic activity driven by cultivation time at the transcriptomic level, providing a theoretical foundation for optimizing the culturing process for this strain and facilitating its application in the green biocontrol of aquaculture diseases.

## Figures and Tables

**Table 1:** Quality assessment and data statistics of transcriptome sequencing samples.

Sample	Raw Reads	Valid Reads	Q30 (%) ^a^	GC (%) ^b^	RIN ^c^
GHZJ-24h-1	16,828,492	16,432,954	97.38	47.89	9.1
GHZJ-24h-2	15,314,700	14,994,218	97.44	47.83	9.4
GHZJ-24h-3	15,351,494	15,023,258	97.50	47.86	9.1
GHZJ-48h-1	15,539,626	14,512,566	97.46	47.34	7.6
GHZJ-48h-2	15,417,828	15,130,942	97.19	46.78	8.4
GHZJ-48h-3	15,368,016	14,442,526	97.58	46.98	8.1

**^a^** Q30, percentage of bases with a Phred quality score greater than 30. ^b^ GC, percentage of guanine and cytosine bases. ^c^ RIN, RNA integrity number.

**Table 2:** Upregulated genes related to secondary metabolite biosynthesis and antimicrobial activity.

Category	ID	log_2_FC	*p*	Swiss-Prot Annotation
Polyketide synthesis	ACNBEW_07325	2.24	3.59 × 10^−18^	PKS PksN
Polyketide synthesis	ACNBEW_07330	2.2	2.72 × 10^−12^	PKS PksL
Polyketide synthesis	ACNBEW_07345	2.04	4.99 × 10^−18^	PKS PksL
Polyketide synthesis	ACNBEW_08635	1.97	7.63 × 10^−21^	PKS PksJ
Polyketide synthesis	ACNBEW_08615	1.97	2.07 × 10^−16^	PKS acyl-carrier protein AcpK
Polyketide synthesis	ACNBEW_07350	1.96	9.89 × 10^−20^	PKS PksL
Polyketide synthesis	ACNBEW_08625	1.93	2.28 × 10^−23^	PKS enoyl-CoA hydratase PksH
Polyketide synthesis	ACNBEW_08630	1.9	4.04 × 10^−23^	PKS enoyl-CoA isomerase PksI
Polyketide synthesis	ACNBEW_07335	1.89	9.95 × 10^−14^	PKS PksN
Polyketide synthesis	ACNBEW_08640	1.88	9.62 × 10^−17^	PKS PksL
Polyketide synthesis	ACNBEW_08600	1.84	6.32 × 10^−31^	PKS malonyl CoA-ACP transacylase BaeC
Polyketide synthesis	ACNBEW_07340	1.72	2.47 × 10^−16^	PKS PksL
Polyketide synthesis	ACNBEW_07355	1.72	1.78 × 10^−10^	PKS PksN
Polyketide synthesis	ACNBEW_08610	1.67	1.88 × 10^−18^	PKS protein BaeE
Polyketide synthesis	ACNBEW_08605	1.6	1.32 × 10^−16^	PKS acyltransferase BaeD
Polyketide synthesis	ACNBEW_08620	1.54	2.84 × 10^−13^	PKS HMG-ACP synthase PksG
Polyketide synthesis	ACNBEW_07320	1.47	7.59 × 10^−9^	PKS protein PksE
Polyketide synthesis	ACNBEW_08645	1.31	1.47 × 10^−11^	PKS PksM
Polyketide synthesis	ACNBEW_08650	1.26	9.72 × 10^−9^	PKS PksN
Thiazole synthesis	ACNBEW_05840	1.34	1.70 × 10^−5^	Thiazole synthase ThiG
Thiazole synthesis	ACNBEW_05825	1.13	2.96 × 10^−2^	Thiazole tautomerase TenI
Thiazole synthesis	ACNBEW_05835	1.94	2.73 × 10^−2^	Sulfur carrier ThiS
Siderophore	ACNBEW_02025	3.39	1.22 × 10^−34^	Petrobactin permease YclN
Siderophore	ACNBEW_02030	2.12	2.69 × 10^−17^	Petrobactin permease YclO
Siderophore	ACNBEW_02035	1.72	6.55 × 10^−14^	Petrobactin ATP-binding YclP
Siderophore	ACNBEW_02040	1.29	2.43 × 10^−7^	Petrobactin-binding YclQ
Siderophore	ACNBEW_14870	1.53	9.12 × 10^−8^	DHB-AMP ligase DhbE
Siderophore	ACNBEW_14875	1.51	1.55 × 10^−6^	Isochorismate synthase DhbC
Siderophore	ACNBEW_14865	1.11	2.15 × 10^−4^	Isochorismatase DhbB
Siderophore	ACNBEW_14880	1.08	1.98 × 10^−3^	DHB dehydrogenase DhbA
Antimicrobial peptide	ACNBEW_06235	2.73	1.62 × 10^−35^	Microcin secretion MchF
Antimicrobial peptide	ACNBEW_18410	2.54	1.75 × 10^−20^	Bacitracin transport BcrA
Antimicrobial peptide	ACNBEW_06230	2.21	1.13 × 10^−22^	Lacticin biosynthesis LcnDR2
Antimicrobial peptide	ACNBEW_17710	1.53	1.09 × 10^−3^	Subtilosin biosynthesis AlbG
Antimicrobial peptide	ACNBEW_01180	1.02	7.87 × 10^−3^	Bacitracin export permease BceB

## Data Availability

The original transcriptome sequencing datasets presented in this study can be found in online repositories. The names of the repository and accession number can be found below: NCBI Sequence Read Archive (SRA) database, accession number PRJNA1474748.
